# Evaluation of Multilocus Sequence Typing of *Cyclospora cayetanensis* based on microsatellite markers

**DOI:** 10.1051/parasite/2019004

**Published:** 2019-01-31

**Authors:** Jessica N. Hofstetter, Fernanda S. Nascimento, Subin Park, Shannon Casillas, Barbara L. Herwaldt, Michael J. Arrowood, Yvonne Qvarnstrom

**Affiliations:** Parasitic Diseases Branch, Division of Parasitic Diseases and Malaria, Center for Global Health, Centers for Disease Control and Prevention Atlanta GA USA

**Keywords:** *Cyclospora cayetanensis*, genotyping, MLST, microsatellites

## Abstract

*Cyclospora cayetanensis* is a human parasite transmitted via ingestion of contaminated food or water. Cases of *C. cayetanensis* infection acquired in the United States often go unexplained, partly because of the difficulties associated with epidemiologic investigations of such cases and the lack of genotyping methods. A Multilocus Sequence Typing (MLST) method for *C. cayetanensis* based on five microsatellite loci amplified by nested PCR was described in 2016. The MLST loci had high variability, but many specimens could not be assigned a type because of poor DNA sequencing quality at one or more loci. We analyzed *Cyclospora*-positive stool specimens collected during 1997–2016 from 54 patients, including 51 from the United States. We noted limited inter-specimen variability for one locus (CYC15) and the frequent occurrence of unreadable DNA sequences for two loci (CYC3 and CYC13). Overall, using the remaining two loci (CYC21 and CYC22), we detected 17 different concatenated sequence types. For four of five clusters of epidemiologically linked cases for which we had specimens from >1 case-patient, the specimens associated with the same cluster had the same type. However, we also noted the same type for specimens that were geographically and temporally unrelated, indicating poor discriminatory power. Furthermore, many specimens had what appeared to be a mixture of sequence types at locus CYC22. We conclude that it may be difficult to substantially improve the performance of the MLST method because of the nucleotide repeat features of the markers, along with the frequent occurrence of mixed genotypes in *Cyclospora* infections.

## Abstract (fr)

*Cyclospora cayetanensis* est un parasite humain transmis par ingestion d’aliments ou d’eau contaminés. Les cas d’infection à *C. cayetanensis* contractés aux États-Unis restent souvent inexpliqués, en partie à cause des difficultés associées aux enquêtes épidémiologiques sur ces cas et de l’absence de méthodes de génotypage. Une méthode de typage génomique multilocus (MLST) pour *C. cayetanensis* basée sur cinq loci de microsatellites amplifiés par PCR imbriquée a été décrite en 2016. Les loci MLST présentaient une grande variabilité, mais de nombreux spécimens n’ont pas pu être attribués à un type en raison de la mauvaise qualité de séquençage de l’ADN pour un ou plusieurs locus. Nous avons analysé des échantillons de selles positifs pour *Cyclospora* prélevés entre 1997 et 2016 auprès de 54 patients, dont 51 aux États-Unis. Nous avons noté une variabilité limitée entre les échantillons pour un locus (CYC15) et la fréquence importante de séquences d’ADN illisibles pour deux loci (CYC3 et CYC13). Globalement, en utilisant les deux loci restants (CYC21 et CYC22), nous avons détecté 17 types différents de séquence concaténées. Pour quatre des cinq groupes de cas épidémiologiquement liés pour lesquels nous avions des échantillons provenant de plus de 1 cas-patient, les échantillons associés au même groupe avaient le même type. Cependant, nous avons également noté le même type pour les spécimens qui n’avaient pas de relation géographique ou temporelle, ce qui indique un faible pouvoir discriminant. En outre, de nombreux spécimens présentaient ce qui semblait être un mélange de types de séquence au locus CYC22. Nous concluons qu’il peut être difficile d’améliorer considérablement les performances de la méthode MLST en raison des caractéristiques de répétition des nucléotides des marqueurs et de la fréquence des génotypes mélangés dans les infections à *Cyclospora*.

## Introduction

*Cyclospora cayetanensis*, the etiologic agent of the enteric illness cyclosporiasis, is a coccidian parasite of humans transmitted via ingestion of contaminated food or water. The two well-established risk factors for US cases of cyclosporiasis are international travel to foci of endemicity (e.g., in the tropics and subtropics) and consumption of fresh produce imported from such areas (e.g., in the context of foodborne outbreaks) [1, 2, 5, 10]. However, the source of infection for many cases of cyclosporiasis acquired in the United States often goes unexplained, in part because of the difficulties associated with epidemiologic investigations of such cases. These challenges are compounded by the lack of genetic typing methods that could facilitate linking cases to each other and to food vehicles and their sources.

Multilocus Sequence Typing (MLST) methods have been used for subspecies identification in epidemiologic investigations of other protozoan parasites. For example, various markers have been used for genotyping *Cryptosporidium parvum*, including markers with variabilities in repeat regions, such as in mini- and microsatellites, as well as markers with single nucleotide polymorphisms (SNPs) [13]. In 2016, Guo et al. described an MLST method for *C. cayetanensis* that incorporated five microsatellite loci amplified by nested PCR [4]. However, the method yielded complete typing information for all five loci for only 34 (53%) of 64 stool specimens because the PCR-generated DNA sequences were often uninterpretable; in addition, for one of the five loci, only one sequence type was identified among the US specimens that were evaluated, regardless of the year and the state in which they were collected. Furthermore, nested PCR is associated with labor-intensive steps and with higher risk for cross-contamination of amplicons than single-step PCR. The aim of our study was to evaluate a modified version of the original MLST method that omitted the nested PCR step and that included only the loci that previously exhibited variability among US specimens.

## Materials and methods

### Stool specimens

We included 58 microscopy-confirmed *Cyclospora*-positive stool specimens in this study. These specimens were collected from 54 patients during 1997–2016; were sent to the Centers for Disease Control and Prevention (CDC) by US health departments and international partners for confirmatory diagnostic testing, for outbreak investigations, or for research purposes; were verified by real-time PCR to be positive for *C. cayetanensis* DNA [11]; and were used in accordance with the CDC-approved human research protocol entitled “Use of coded specimens for *Cyclospora* genomics research.” To be suitable for analysis and therefore for inclusion in this study, stool specimens had to have been received by CDC either unpreserved, suspended in non-nutritive media (e.g., Cary-Blair transport medium), preserved in alcohol-based fixatives (e.g., TOTAL-FIX, Medical Chemical Corporation, Torrance, CA), or preserved in 2.5% aqueous potassium dichromate. Among the specimens that were suitable for analysis, we prioritized specimens from patients known by CDC to be epidemiologically linked to a cluster of cases or an outbreak of cyclosporiasis (22 such specimens/patients); specimens, if still available, for which MLST typing had been conducted by Guo et al. [4] (five such specimens/patients); and specimens collected from patients who provided more than one specimen (a total of seven such specimens from three patients). We also included 21 other stool specimens collected from 21 US patients during 2013–2016, as well as three specimens from countries in which cyclosporiasis is known to be endemic (i.e., one specimen/patient each from China, Guatemala, and Indonesia).

### DNA extraction

Stool specimens washed free of preservative by centrifugation were diluted with phosphate-buffered saline (pH 7.2) to form a thick slurry. DNA was extracted using the Universal Nucleic Acid Extraction (UNEX)-based method described by Qvarnstrom et al. [11]. Purified DNA was stored at 4 °C.

### PCR amplification

We did not evaluate the CYC15 microsatellite locus in this study because only one sequence type was identified at this locus among the US specimens analyzed in the original study [4]. We amplified the other four loci (CYC3, CYC13, CYC21, and CYC22), initially using nested PCR as described previously [4]. We performed single-step PCR using only the inner primers (i.e., the primers named F2 and R2 [4]), at 250 nMol/L, with AmpliTaq Gold 360 Master Mix (Applied Biosystems, Foster City, CA) and 1 μL DNA in a total volume of 40 μL. PCR primer sequences are listed in Supplementary Table S1. We used the following amplification strategy: denaturation at 94 °C for 10 min, followed by 40 cycles of 94 °C for 45 s; an annealing temperature of 55 °C (for loci CYC3, CYC21, and CYC22) or 58 °C (for locus CYC13) for 1 min; 72 °C for 45 s; and a final extension at 72 °C for 7 min. PCR products were visualized on 1.5% agarose gels stained with ethidium bromide, were purified using Monarch PCR DNA Cleanup Kit (New England Biolabs, Ipswich, MA), and were stored for up to 3 days at 4 °C until sequenced.

### DNA sequencing

PCR products were Sanger sequenced in both directions using the amplification primers and the BigDye Terminator v3.1 chemistry (Applied Biosystems, Foster City, CA). Unincorporated dye terminators were removed using a DyeEx 2.0 Spin Kit (QIAGEN, Hilden, Germany). DNA sequencing reads were analyzed on an ABI PRISM 3130xl Genetic Analyzer (Applied Biosystems, Foster City, CA).

### Analysis of DNA sequence data

Sanger-sequencing chromatograms were imported into Geneious 10 [7] and aligned with reference sequences from GenBank using the MUSCLE tool [3]. Reference sequences with accession numbers KP723491 through KP723494 and KY770752 through KY770754 for CYC3, KP723495 through KP723503 and KY770764 through KY770769 for CYC13, KP723507 through KP723514 and KY770770 through KY770776 for CYC21, and KP723515 through KP723518 and KY770777 for CYC22 were used to assign sequence types. New sequences were deposited in GenBank with accession numbers MF155932 for CYC21-C16 and MG972882 for CYC22-C6.

## Results

### Evaluation of the nested PCR-based MLST methodology

For our initial evaluation of the four pertinent MLST loci (i.e., CYC3, CYC13, CYC21, and CYC22), we used 25 (of the 58 total) stool specimens, each from a different patient, and amplified DNA using the nested PCR approach. These 25 specimens included, among others, 12 collected from patients linked to seven separate US clusters/outbreaks (in 1997, 2001, 2014, and 2015) and one specimen each collected in Guatemala and Indonesia (Supplementary Table S2). For the CYC3 locus, only 17 (68%) of the 25 specimens could be assigned a type, the DNA from five specimens failed to amplify, and the DNA sequences were unreadable for three specimens. Because of this high failure rate, as well as low variability (we observed only two sequence types), we excluded CYC3 from further evaluation. The CYC13 locus was amplified from 21 specimens (84%), but the quality of the sequences in the terminal 130-base-pair region was poor for all but one specimen. Because this terminal region of CYC13 includes SNPs and insertions important for type differentiation, we were unable to assign reliable sequence types for this locus and we excluded it from further evaluation.

**Figure 1:**
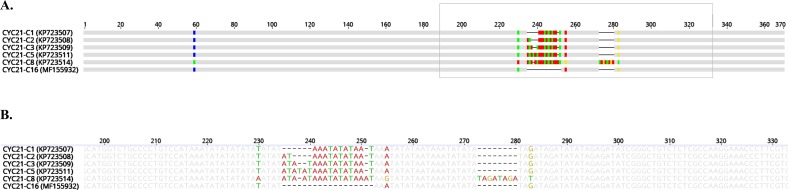
CYC21 sequence types. (A) Nucleotide polymorphisms among five of the previously identified CYC21 sequence types [4] and the newly identified sequence type C16. (B) Sequence detail of the boxed area in Panel A.

**Figure 2:**
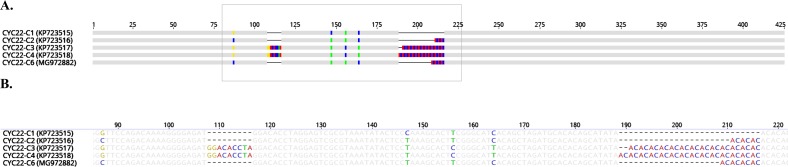
CYC22 sequence types. (A) Nucleotide polymorphisms among the four previously identified CYC22 sequence types [4] and a newly identified sequence type C6 (sequence type C3mixed is not shown because C3mixed and C4 had identical consensus sequences). (B) Sequence detail of the boxed area in Panel A.

We observed four CYC21 types (C2, C3, C5, and a new type not previously published, which we designated C16) among the 24 specimens (96%) from which DNA was successfully amplified via nested PCR (Fig. 1). The CYC22 locus was amplified via nested PCR from 23 specimens (92%). We observed four different types (C1, C2, C3, and C4) among 14 of these specimens, whereas the other nine specimens had sequencing results that we interpreted as a mixture of sequence types C3 and C4 (these two types differ by only one set of dinucleotide AT repeats; see Fig. 2). In contrast to Guo et al., who reported this result as “noisy” and not informative, we decided, for the purposes of this study, to refer to this type as C3mixed. The mixed-type result was reproducible using both nested and single-step PCR and was the most common result at locus CYC22 among the specimens analyzed.

### Sequence typing using loci CYC21 and CYC22 amplified by single-step PCR

Based on the results we obtained using nested PCR, we included CYC21 and CYC22 in an expanded study using single-step PCR to amplify DNA from a total of 58 specimens (including the 25 specimens initially analyzed by nested PCR, as described above) from 54 patients. Overall, for the 25 specimens we analyzed by both nested PCR and single-step PCR, the results were the same with both techniques; we did not detect a change in the quality of the DNA sequences when we omitted the nested step. Nor did more specimens fail to amplify without the nested step, probably because all of the specimens in this study tested positive by microscopy and, therefore, contained relatively high numbers of oocysts. Supplementary Table S2 summarizes the typing results and available epidemiologic information for all 58 specimens.

In this study, we observed six sequence types at locus CYC21 (C1, C2, C3, C5, C8, and C16; see Fig. 1) and six sequence types at locus CYC22 (C1, C2, C3, C3mixed, C4, and a previously unpublished type we designated C6; see Fig. 2). The most commonly identified sequence type at locus CYC21 was C2 and at locus CYC22 was C3mixed (Table 1). Nine specimens (16%) from eight patients did not yield readable sequence data for either or both of the two loci. Concatenation of the CYC21 and CYC22 loci for the other 49 specimens, which were from 46 patients, resulted in 17 different type combinations (Table 2), each of nine of which we detected in single specimens, such as type C1-C6 (for the specimen from China) and type C2-C3 (for the specimen from Indonesia). The most commonly identified concatenated sequence type was C2-C3mixed, which we detected in specimens from 13 US patients, including 11 in Texas in 3 years (2013, 2015, and 2016) and two in South Carolina in 2014. The second most common sequence type was C2-C1, which we detected in specimens from six US patients, including patients from Rhode Island in 1997 (one), New York in 2001 (one), Texas in 2013 (one), Texas in 2014 (one), and Texas 2016 (two).

**Table 1:** Sequence types identified in 58 *Cyclospora*-positive specimens from 54 patients^1^.

Sequence type		Number of patients (%)
CYC21 locus	C1	1 (2)
	C2	30 (56)
	C3	6 (11)
	C5	6 (11)
	C8	2 (4)
	C16	4 (7)
	Not amplified or readable	5 (9)
	Total	54 (100)
CYC22 locus	C1	10 (19)
	C2	9 (17)
	C3	1 (2)
	C3mixed	23 (43)
	C4	3 (6)
	C6	1 (2)
	Not amplified or readable	7 (13)
	Total	54 (100)

1 For the three patients from whom more than one specimen was analyzed, the same results were obtained for each of the specimens.

**Table 2:** Concatenated sequence types observed among 49 stool specimens (from 46 patients) with typing results for both loci CYC21 and CYC22.

Concatenated sequence type (CYC21-CYC22)	Number of specimens	Epidemiologic linkage to a cluster or outbreak (vehicle and source, if both were identified)^1^
C1-C6	1	No
C2-C1	1	Multistate outbreak in 1997 (raspberries from Guatemala) [6]
	1	Florida outbreak in 2001 [2]
	2	Texas restaurant-associated cluster in 2016 [2]
	2	No
C2-C2	3	Maine temporospatial cluster in 2014
	2	No
C2-C3	1	No
C2-C3mixed	2	South Carolina temporospatial cluster in 2014 [2]
	11	No
C2-C4	1	Texas restaurant-associated cluster 2014-A (cilantro from Mexico) [2]
	3 (same patient)	No
C3-C1	1	No
C3-C2	1	Michigan conference-associated cluster in 2014 [2]
C3-C3mixed	3	Texas business-associated cluster in 2015
C3-C4	1	Texas restaurant-associated cluster 2014-B
C5-C2	1	No
C5-C3mixed	5	No
C8-C1	1	No
C8-C2	1	No
C16-C1	1	Michigan conference-associated cluster in 2014 [2]
C16-C2	2 (same patient)	No
C16-C3mixed	2	Michigan conference-associated cluster in 2014 [2]

1 The epidemiologic information provided in the table reflects data submitted to CDC as part of surveillance or outbreak-related activities for cyclosporiasis. The terminology temporospatial cluster is used here for cases that were not linked to a particular establishment or event but were temporally and geographically clustered.

As noted in the Materials and Methods section, three (of 54) patients provided more than one specimen (Supplementary Table S2). One of those patients provided three specimens, all of which had sequence type C2-C4; one patient provided two specimens, both of which had sequence type C16-C2; and one other patient provided two specimens, neither of which produced readable sequence data for either locus. For the five specimens (one per patient) included in this study that had been analyzed previously by Guo et al. (Supplementary Table S2), we obtained the same results that they reported, with the key exception that we assigned a sequence type (i.e., C3mixed) for locus CYC22 for the four specimens for which they had considered the results uninterpretable.

This study included 22 specimens from 22 patients with known associations with case clusters or outbreaks. Four of these specimens did not yield readable sequence data for one or both of the typing loci. The other 18 specimens were associated with nine separate clusters/outbreaks (Table 3). However, both the food vehicle of infection and its source were identified for only two of these nine clusters/outbreaks (raspberries from Guatemala for the multistate outbreak in 1997 [6] and cilantro from Mexico for restaurant-associated cluster A in Texas in 2014 [2]). The study included specimens from more than one patient per cluster for only five of the nine clusters (Maine temporospatial cluster in 2014, Michigan conference-associated cluster in 2014, South Carolina temporospatial cluster in 2014, Texas business-associated cluster in 2015, and Texas restaurant-associated cluster in 2016). For four of these five clusters, the specimens from patients associated with the same cluster had the same concatenated sequence type. However, the fifth cluster (Michigan conference-associated cluster in 2014) encompassed three different MLST types among the specimens from four patients with readable sequence data for both of the typing loci. If the CYC21 sequence result (i.e., C2) is taken into account for a Michigan cluster-associated specimen/patient without sequence data for locus CYC22, this cluster encompassed four concatenated sequence types among five patients (Supplementary Table S2).

**Table 3:** Epidemiologic information^1^ and typing results for 18 stool specimens (with typing data for both loci CYC21 and CYC22) from 18 patients associated with case clusters or outbreaks.

Collection location^2^ and year	Epidemiologic linkage to a cluster or outbreak (vehicle and source, if both were identified)^3^	Specimen ID	International travel during 2-week period before symptom onset^4^	Sequence type	
				Locus CYC21	Locus CYC22
Rhode Island 1997	Multistate outbreak (raspberries from Guatemala) [6]	HCRI001_97	No	C2	C1
New York 2001	Florida outbreak [2]	HCNY016_01	No	C2	C1
Maine 2014	Maine temporospatial cluster	HCME548_14	No	C2	C2
		HCME550_14	No	C2	C2
		HCME552_14	No	C2	C2
Michigan 2014	Michigan conference-associated cluster [2]	HCMI029_14	No	C16	C1
		HCMI030_14	Unknown	C3	C2
		HCMI039_14	Unknown	C16	C3mixed
Pennsylvania 2014		HCPA556_14	No	C16	C3mixed
South Carolina 2014	South Carolina temporospatial cluster [2]	HCSC052_14	No	C2	C3mixed
		HCSC053_14	No	C2	C3mixed
Texas 2014	Texas restaurant-associated cluster 2014-A (cilantro from Mexico) [2]	HCTX543_14	No	C2	C4
	Texas restaurant-associated cluster 2014-B	HCTX592_14	No	C3	C4
Texas 2015	Texas business-associated cluster	HCTX204_15	Cozumel, Mexico	C3	C3mixed
		HCTX205_15	No	C3	C3mixed
		HCTX538_15	No	C3	C3mixed
Texas 2016	Texas restaurant-associated cluster [2]	HCTX471_16	No	C2	C1
		HCTX474_16	No	C2	C1

1 The epidemiologic information provided in the table reflects what was submitted to CDC as part of surveillance or outbreak-related activities for cyclosporiasis.

2 The collection location was not necessarily the same as the place of exposure to *C. cayetanensis*.

3 The terminology temporospatial cluster is used here for cases that were not linked to a particular establishment or event but were temporally and geographically clustered.

4 Because US patients with a history of international travel may have spent part of the 2-week period before illness onset in the United States, the specified travel destination is not necessarily where they became infected.

## Discussion

We evaluated a modified typing methodology for *C. cayetanensis* based on microsatellite markers*.* The original MLST method described by Guo et al. [4] incorporated five loci that were amplified using a nested PCR assay. We excluded one locus (CYC15) that, in general, did not discriminate among stool specimens from US patients. We also excluded loci CYC3 and CYC13 (information rich but unreliable) because we were unable to obtain readable sequences for a high proportion of the specimens. We also simplified the approach by removing the nested PCR step. This did not have any discernable effect on the typing results in our study, in which we used microscopy-positive stool specimens. However, use of the two-step nested procedure may be important to achieve adequate sensitivity for stools or environmental samples that may contain few oocysts.

The two previously published articles of the five-loci *C. cayetanensis* MLST also reported substantial problems with low-quality sequence results. Guo et al. obtained successful DNA amplification and readable sequences for all five loci for only 34 (53%) of 64 *C. cayetanensis*-positive specimens [4]. For 26 specimens (41%), DNA was successfully amplified but the investigators classified the sequences for one or more loci as unreadable, including 11 specimens with “noisy” results at locus CYC22 (seven specimens collected in Texas in 2013, one from New York in 1997, one from New York in 1998, and two from China in 2009). Three of the specimens with “noisy” CYC22 results (the two from New York and one of the two from China) also had “noisy” results for CYC3. In retrospective analyses we conducted of the CYC22 sequence data for seven (the specimens from Texas in 2013) of these 11 specimens, all seven had the pattern we referred to as C3mixed. Assuming that all 11 “noisy” CYC22 results could be reclassified as type C3mixed, the success rate (for all five loci) in the study by Guo et al. would increase to 42 (66%) of 64 specimens. Because Li et al. [8], who obtained readable sequences for all five loci for only 45 (59%) of 76 *C. cayetanensis*-positive specimens (56 [74%] of 76 specimens for the CYC22 locus), did not distinguish failed PCR amplification from unreadable DNA sequences, we do not know whether and to what extent the occurrence of the C3mixed type at locus CYC22 contributed to the low success rate.

The reasons for the uninterpretable sequences from the MLST markers have not been explored. Guo et al. proposed that the main reason for their “noisy” sequences was the presence of PCR products with different repeat lengths. In our study, we confirmed this explanation for the CYC22 marker, where the pattern of overlapping signals for the C3mixed type indicated the presence of two PCR products that differed in length by two nucleotides. The unreadable sequences encountered for the other loci were more difficult to explain, perhaps because more than two different amplicon lengths were involved. Nonspecific PCR amplification and suboptimal primer binding are other possibilities because, to our knowledge, the specificity and sensitivity of the PCR primers have not been evaluated. The MLST method was developed based on the draft genome for only one isolate of *C. cayetanensis* (the first full draft genome available). A search for the MLST PCR primer sequences in the 20 draft genomes of *C. cayetanensis* available in the NCBI genome database as of December 2018 found that the primer sequences are either missing or contain mismatches in zero, two, three, or eight genomes for CYC3, CYC13, CYC21, and CYC22, respectively (data not shown). Therefore, the amplification and sequencing efficiencies might be improved by re-designing the PCR primers. For CYC13, moving the PCR primer binding sites downstream would also allow for improved sequencing of the variable positions near the 3′ end of the current amplicon.

The lack of a reliable typing method for *C. cayetanensis* has contributed to the challenges associated with detecting and investigating outbreaks of cyclosporiasis – i.e., to linking cases to each other as well as to particular food vehicles of infection and to the sources of those vehicles. In this study, only 22 (of 58 total) specimens were from patients with known epidemiologic associations with case clusters/outbreaks, only 18 such specimens (one per patient) yielded readable sequence data for both typing loci we evaluated (CYC21 and CYC22), and only 14 such specimens were linked to clusters for which we had more than one specimen/patient per cluster. For four of the five such clusters, the specimens/patients linked to the same cluster had the same concatenated sequence type. However, the Michigan conference-associated cluster in 2014 encompassed multiple sequence types. No vehicle of infection was identified in the epidemiologic investigation of that cluster, which was associated with multiday events; whether the various sequence types were associated with the same exposure is not known. However, the occurrence of multiple MLST types among patients from the same cyclosporiasis-endemic community or area in various countries was documented by Guo et al. [4].

An efficient genotyping method must group related specimens together and distinguish unrelated specimens. Important criteria for the performance of a genotyping method include typeability (the ability of the method to assign a type to all specimens), discriminatory power (the ability of the method to assign different types to unrelated specimens), epidemiologic concordance, and reproducibility [12]. Previous studies of the five-loci MLST method found that it had adequate discriminatory power (e.g., 25 MLST types among 34 specimens in the first study [4], resulting in a diversity index of 0.97), but the typeability was low (53–59%) because of the problem with unreadable sequences. Epidemiologic concordance and reproducibility were not addressed. On the other hand, the two-loci method evaluated in this study had better typeability (84%); but the diversity index was reduced to 89%, indicating lower discriminatory power (e.g., we observed the same concatenated sequence type C2-C1 in specimens from patients not expected to be related on the basis of geographic or temporal criteria). For specimens with readable sequences, the MLST results were reproducible and consistent with available epidemiologic data for four of five clusters.

Because *C. cayetanensis* is a eukaryotic parasite that undergoes sexual reproduction within the gut of infected humans, the occurrence of heterozygous sequences and mixed genotypes is expected. Therefore, Sanger sequencing may not be ideal for genotyping this organism. An alternative approach could be to perform Next Generation Sequencing (NGS). Deep amplicon sequencing using NGS can resolve mixed genotypes and thereby may increase the number of specimens with interpretable sequence data. However, the MLST loci are based in part on repeat-length differences in microsatellites, which are difficult to analyze from NGS data. A common approach when using mini- and microsatellites for genotyping is to use fragment-length analysis instead of amplicon sequencing [9]. However, fragment-length analysis cannot be applied to the MLST loci developed for *C. cayetanensis* because the repeat units are too short (only two or three base pairs) and all loci include SNPs and insertions/deletions in addition to the repeats.

In summary, the original MLST technique failed to assign a type to a high proportion of specimens, mostly because of unreadable sequences [4, 8]. A revised method based on the two loci with the best sequencing results (CYC21 and CYC22) had better typeability and good epidemiologic concordance but could not reliably differentiate unrelated specimens. We conclude that it may be difficult to substantially improve the performance of the MLST method because of the nucleotide repeat features of the markers along with the frequent occurrence of mixed genotypes in *Cyclospora* infections.

## Supplementary material

Supplementary materials are available at https://www.parasite-journal.org/10.1051/parasite/2019004/olm.
