# Supplementary material for: Evaluation of Multilocus Sequence Typing of Cyclospora cayetanensis based on microsatellite markers
Source: Parasite. 2019 Jan 31;26:3. doi: 10.1051/parasite/2019004 (PMC6354607; doi:10.1051/parasite/2019004)
Supplement: Supplementary file 1 [file parasite-26-3-olm.pdf]

Supplementary Table S1. PCR primer sequences used to amplify the MLST markers CYC3, CYC13, CYC21 and CYC22 [4].

locus	Outer primer sequences (5' to 3')	Inner primer sequences (5' to 3')
CYC3	F1: GAAGATGAAGCGTTGGTACG<br>R1: TACCGCTGCTGGAGTGCAT	F2: TTGTGCATGGCACCCAATGC<br>R2: CCAGACAGTAGTTCGTGTCTT
CYC13	F1: TTGGAGCAGGACGAGTTTCG<br>R1: ATGGAAGCGGCTATGAAATTGG	F2: CCTCGGAGTCCTCTGAGTG<br>R2: AGCCGTCGCAGTGTGTAGCA
CYC21	F1: TAGTGGCGACTGCGACATG<br>R1: GCACCTTGCTGATGAGGCA	F2: CTAAGGCTGTCTTGAGCGG<br>R2: CGCCCACATGCTTCGTATAC
CYC22	F1: CACTATGCCGTGTGACACGT<br>R1: GTAGATTTGCAAGAACTCATGCTA	F2: ATAGTATTCAGGCGCAAACTAAG<br>R2: GAGGCTTTCCAAAGGTCTAGTT

Supplementary Table S2. Epidemiologic information[1] and typing results for 58 stool specimens from 54 patients

Collection location[2] and year	Epidemiologic linkage to a cluster or outbreak (vehicle and source, if both were identified)[3]	Specimen ID	International travel during 2-week period before symptom onset[4]	Sequence type	
				Locus CYC21	Locus CYC22
Guatemala 1997	No	HCGM011_97[5]	Not applicable	C2	-
Rhode Island 1997	Multistate outbreak (raspberries from Guatemala) [6]	HCRI001_97[5]	No	C2	C1
New York 2001	Florida outbreak [2]	HCNY016_01[5]	No	C2	C1
China 2011	Unknown	CHN_HEN01	Not applicable	C1	C6
Florida 2013	Unknown	HCFL747_13[5]	Unknown	C5	C2
Iowa 2013[6]	Unknown	HCIA002_13[5]	Unknown	C5	C3mixed
	Unknown	HCIA003_13[5]	Unknown	C5	C3mixed
	Unknown	HCIA004_13	Unknown	C5	C3mixed
	Unknown	HCIA006_13	Unknown	C5	C3mixed
Texas 2013[6]	Unknown	HCTX119_13[5] (41405)[7]	Unknown	C2	C1
	Unknown	HCTX135_13[5] (41407)[7]	Unknown	C2	C3mixed
	Unknown	HCTX363_13[5] (41409)[7]	Unknown	C2	C3mixed
	Unknown	HCTX141_13[5] (41410)[7]	Unknown	C2	C3mixed
	Unknown	HCTX120_13[5] (41411)[7]	Unknown	C5	C3mixed
Indonesia 2014	Unknown	HCJK001_14[5]	Not applicable	C2	C3

Maine 2014	Maine temporospatial cluster	HCME548_14[5]	No	C2	C2
		HCME550_14[5]	No	C2	C2
		HCME552_14[5]	No	C2	C2
Michigan 2014	Michigan conference-associated cluster [2]	HCMI029_14[5]	No	C16	C1
		HCMI030_14[5]	Unknown	C3	C2
		HCMI031_14	No	C2	-
		HCMI039_14[5]	Unknown	C16	C3mixed
		HCMI040_14	No	-	-
Pennsylvania 2014		HCPA556_14	No	C16	C3mixed
South Carolina 2014	South Carolina temporospatial cluster [2]	HCSC052_14[5]	No	C2	C3mixed
		HCSC053_14[5]	No	C2	C3mixed
		HCSC054_14	No	C2	-
Texas 2014	No	HCTX535_14[5]	No	C3	C1
	No (two specimens from same patient)	HCTX539_14	Cancun, Mexico	C16	C2
		HCTX540_14[5]	Cancun, Mexico	C16	C2
	Texas restaurant-associated cluster 2014-A (cilantro from Mexico) [2]	HCTX543_14[5]	No	C2	C4
	No (two specimens from same patient)	HCTX551_14	No	-	-
		HCTX552_14	No	-	-
	No (three specimens from same patient)	HCTX554_14	No	C2	C4
		HCTX555_14	No	C2	C4

		HCTX556_14[5]	No	C2	C4
	No	HCTX560_14	Puerto Vallarta, Mexico	C2	C2
	Texas restaurant-associated cluster 2014-B	HCTX592_14	No	C3	C4
	No	HCTX697_14	Mexico (region not specified)	C2	C1
Texas 2015	Texas business-associated cluster	HCTX204_15	Cozumel, Mexico	C3	C3mixed
		HCTX205_15	No	C3	C3mixed
		HCTX538_15	No	C3	C3mixed
	No	HCTX227_15	Playa del Carmen, Mexico	C2	C3mixed
	No	HCTX230_15	Riviera Maya, Mexico	C2	C2
	No	HCTX551_15	Cancun, Mexico	C2	C3mixed
	Texas restaurant-associated cluster	HCTX353_15[5]	No	-	-
	No	HCTX355_15	Riviera Maya, Mexico	C2	C3mixed
	No	HCTX199_15	Playa del Carmen, Mexico	C8	C1
Virginia 2015	Unknown	HCVA002_15	Unknown	-	C1
TX 2016	No	HCTX500_16	No	-	-
	No	HCTX503_16	Cancun, Mexico	C8	C2
	No	HCTX504_16	Playa del Carmen, Mexico	C2	C3mixed
	No	HCTX462_16	Mexico (region not specified)	C2	C3mixed

	No	HCTX468_16	Cancun, Mexico	C2	C3mixed
	Texas restaurant-associated cluster [2]	HCTX471_16	No	C2	C1
		HCTX474_16	No	C2	C1
	No	HCTX475_16	Cancun, Mexico	C2	C3mixed
	No	HCTX493_16	Puerto Morelos, Mexico	C2	C3mixed

[1]The epidemiologic information provided in the table reflects what was submitted to CDC as part of surveillance or outbreak-related activities for cyclosporiasis.

[2]The collection location was not necessarily the same as the place of exposure to *C. cayetanensis*.

[3]The terminology temporospatial cluster is used here for cases that were not linked to a particular establishment or event but were temporally and geographically clustered.

[4]Because U.S. patients with a history of international travel may have spent part of the 2-week period before illness onset in the United States, the specified travel destination is not necessarily where they became infected.

[5]Specimen was included in the nested-PCR evaluation.

[6]Although foodborne outbreaks of cyclosporiasis were documented in Iowa and Texas in 2013 [1, 2], CDC did not receive information regarding whether the specimens listed here were obtained from patients with outbreak- or travel-associated cases.

[7]The 5-digit number in parentheses constitutes the specimen ID used by Guo et al. [4].
